# halSynteny: a fast, easy-to-use conserved synteny block construction method for multiple whole-genome alignments

**DOI:** 10.1093/gigascience/giaa047

**Published:** 2020-05-28

**Authors:** Ksenia Krasheninnikova, Mark Diekhans, Joel Armstrong, Aleksei Dievskii, Benedict Paten, Stephen O’Brien

**Affiliations:** 1 Computer Technologies Laboratory, School of Translational Information Technologies, ITMO University, 49 Kronverkskiy Pr., St. Petersburg 197101, St. Petersburg, Russian Federation; 2 Wellcome Trust Sanger Institute, Wellcome Trust Genome Campus, Hinxton CB10 1SA, UK; 3 UC Santa Cruz Genomics Institute, Santa Cruz, CA, USA; 4 Guy Harvey Oceanographic Center, Halmos College of Natural Sciences and Oceanography, Nova Southeastern University, 8000 North Ocean Drive, Ft Lauderdale, FL 33004, USA

**Keywords:** Synteny blocks, genome alignments, comparative genomics, HAL format

## Abstract

**Background:**

Large-scale sequencing projects provide high-quality full-genome data that can be used for reconstruction of chromosomal exchanges and rearrangements that disrupt conserved syntenic blocks. The highest resolution of cross-species homology can be obtained on the basis of whole-genome, reference-free alignments. Very large multiple alignments of full-genome sequence stored in a binary format demand an accurate and efficient computational approach for synteny block production.

**Findings:**

halSynteny performs efficient processing of pairwise alignment blocks for any pair of genomes in the alignment. The tool is part of the HAL comparative genomics suite and is targeted to build synteny blocks for multi-hundred–way, reference-free vertebrate alignments built with the Cactus system.

**Conclusions:**

halSynteny enables an accurate and rapid identification of synteny in multiple full-genome alignments. The method is implemented in C++11 as a component of the halTools software and released under MIT license. The package is available at https://github.com/ComparativeGenomicsToolkit/hal/.

## Introduction

Conserved synteny blocks provide a conceptual framework for the analysis of interspecies homology. Originally, the notion of synteny stems from the area of cell genetics, where it was defined as the co-location of ≥2 homologous genes on the same chromosome [1]. This term has been adopted by the comparative genomics field to refer to contiguously aligned regions that preserve order and orientation of the alignment while allowing for micro-rearrangements within the syntenic region [2]. These genomics approaches introduce some quantitative properties of blocks, such as the size of blocks and resolution of synteny in bases.

There are a number of existing tools designed for finding synteny blocks. The GRIMM-Synteny [2] algorithm reconstructs an anchor graph from the predefined set of homologous hits shared by genomes, which can be local pairwise alignments or orthologous genes. The chains-and-nets algorithm [3] introduces a novel BLASTZ scoring scheme for identification of alignment anchors between 2 species. A chained alignment is built over an ordered sequence of traditional pairwise nucleotide alignments; then the set of chains is processed into nets using the chains with the highest score. DAGchainer [4] implements a directed acyclic graph (DAG)-based approach over predefined pairs of gene anchors. Satsuma [5] describes application of the fast Fourier transform algorithm over the signal represented by the nucleotide multiplication pattern. MCScanX [6] operates over the gene sets and applies tuned scoring schemes in the dynamic programming algorithm over chains of pairwise gene alignments. i-ADHoRe [7] introduces homology matrices to resolve homology among tandem replications of genes; furthermore, the Needleman-Wunsch algorithm is applied for detection of collinearity. SynChro [8] operates over reciprocal best hits (RBH) obtained from BLASTP alignments for reconstruction of the synteny block backbones. In comparison to GRIMM-Synteny, which allows local disruptions of synteny measured in genomic intervals, SynChro allows for an unlimited number of non-RBH genes but preserves the required number of intermediate RBH genes between first and last gene in a synteny block. The DRIMM-Synteny [9] algorithm implements application of A-Bruijn graphs over a set of predefined anchors. Analogously to GRIMM-Synteny it can be, e.g., local alignments or pairs of similar genes. In contrast to GRIMM-Synteny it provides a resistance against unwanted synteny disruption when a search is performed over multiple genomes and homologous anchors may be absent in a small proportion of the genomes analyzed. The SyMAP [10] algorithm computes the raw hits between nucleotide sequences of a pair of genomes, which are then clustered and filtered using the optional gene annotation. CYNTENATOR [11] uses phylogenetic information and performs progressive alignment of the gene order among multiple genomes.

These tools all require various data formats, which must be derived from the alignment, such as a predefined set of homologous genomic markers, or genome alignment blocks, each being a sequence of aligned bases that is contiguous in each of the genomes represented by the block. Many of them also require a rigorous and reliable annotation of orthologous genes. With halSynteny, the alignment is the only required input.

With the increased availability of large-scale computing facilities, multiple-vertebrate whole-genome alignment is now tractable. Multi-species genome alignments are a useful tool for analysis of species homology in large-scale comparative genomic projects [12,13]. One of the state-of-the-art tools [14–16] is Progressive Cactus [17,18] which produces reference-free all-to-all genome alignments.

By producing a single, reference-free multiple alignment, Cactus allows synteny block reconstruction between any 2 genomes without reference bias, directly from the HAL representation. Here we present halSynteny, a tool that implements a DAG-based algorithm for identification of synteny blocks directly from HAL alignment and reporting synteny blocks in PSL format [19].

## Methods

### Algorithm

We describe a heuristical algorithm that operates on a pair of selected genome assemblies in the HAL multiple alignment. A synteny block is a sequence of local alignments that in each genome maintain the following properties: (i) are on 1 chromosome, (ii) do not overlap, (iii) are on the same strand, and (iv) have chromosome sequence coordinates that are either monotonically increasing (for positive strand) or decreasing for negative strand [2]. The set of synteny blocks over a pair of genomes is parameterized by the lower bound of minimal block length *b*_min_ and maximal distance *d*_max_ between 2 sequential anchoring alignment blocks. The pair (*b*_min_, *d*_max_) can be regarded as a resolution of the synteny block.

Each gapless alignment block between the pair of genomes is represented with the start and end positions on the chromosomes, along with the strand. Duplications are expressed as overlapping alignment blocks. Presume that there are alignment hits in genome A that can be ordered by genomic coordinates as *p*_1_⋅⋅⋅*p_i_*, ⋅⋅⋅*p_n_* and in genome B as *u*_1_⋅⋅⋅*u_i_*, *u*_*i* + 1_, ⋅⋅⋅*u*_*n* + 1_, and there are alignments present among segments *p_l_* and *u_l_* for *l* ∈ 1⋅⋅⋅*i p_k_* and *u*_*k* + 1_ for *k* ∈ *i*⋅⋅⋅*n*. Then the synteny blocks between these 2 genomes can overlap and contain the following pairs of segments: (*p_i_*, *u_i_*) for *l* ∈ 1⋅⋅⋅*i* and (*p_k_*, *u*_*k* + 1_) for *k* ∈ *i*⋅⋅⋅*n*.

The set of graph vertices *V* is formed by alignment blocks. Vertex *v_j_* is defined syntenic to *v_i_* if each genome maintains the same order and orientation, their corresponding genomic coordinates do not overlap, and the genomic distance in either genome between *v_i_* and *v_j_*, *d_ij_*, does not exceed the maximal distance *d*_max_ defined by the synteny resolution. The set of graph edges *E* is formed by all pairs (*v_i_*, *v_j_*) such that *v_j_* is syntenic to *v_i_*. This results in the set of DAG subgraphs corresponding to synteny regions in the graph of alignment blocks. The desirable set of synteny blocks would contain as many continuous synteny blocks as possible covering as much of both genome sequences as possible. To achieve this goal we build the graph *G* = <*V, E*> and apply the following algorithm:

1. Initialize weight labels of vertices and edges:
  - Initialize the weight of each vertex $w_{v_i}$ as the absolute value of the difference between start and end coordinates in the target of query genomes, which is called the size of the corresponding alignment block.
  - The weight *w_i_* of each edge coming into a vertex *v_i_* is defined as the initial weight of the vertex *v_i_*.
2. Traverse the vertices in topological order.:
  - For each vertex *v_i_* consider all the edges (*v_i_*, *v_j_*) and for all incident vertices *v_j_* calculate the candidate weight update, defined as the weight of an edge coming into *v_j_* plus the weight of the preceding vertex *v_i_*.
  - If the candidate weight is greater than the current weight of *v_j_*, replace the weight of *v_j_* with this value.
  - If *w_j_* was updated, store the parent vertex *v_i_* for backtracking.
3. Find the vertex with maximal weight and trace back using stored previous vertices.
4. If the path built from vertices obtained at Step 3 is at least as long as the predefined minimal block length (defined by resolution), then remove them from the vertex set and store this path as a synteny block; else stop execution. If *V* is not empty, then go to Step 3.

As a result, we construct a set of paths that are possibly overlapping in genomic coordinates, so that each path covers as much of each genome as possible.

### Evaluation of results

To assess the accuracy of this algorithm, we constructed synteny blocks between the domestic cat (FelCat 8.0) and domestic dog (CanFam 3.1) genomes based on Progressive Cactus alignment of these genomes, together with the human genome (GRCh38) as an outgroup.

**Figure 1:**
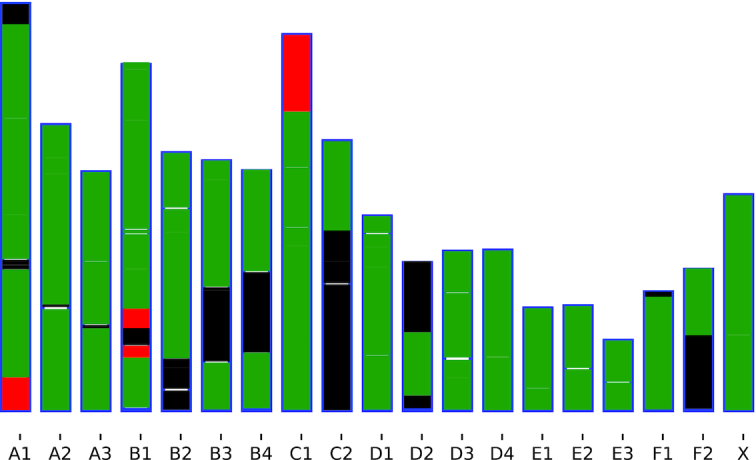
Comparison of produced synteny blocks between halSynteny with parameters –maxAnchorDistance 1000000 (1 Mb), –minBlockSize 1000000 (1 Mb), and results obtained by chromosomal painting [20] of domestic cat chromosomes with those of domestic dog. Blue contour depicts the borders of chromosomes. Green segments indicate regions where both methods have identified the same homologous regions in the dog genome (80.9% of cat genome). Red indicates regions where different homologous dog regions are identified (3.4%). Black stretches indicate regions not covered by chromosome painting where halSynteny has produced synteny blocks (14.6%). The white regions correspond to segments with no halSynteny blocks that may be covered partly by chromosome painting (0.01%). Because the chromosomal painting approach is not bound to any assembly and does not produce any genomic coordinates, 2 assignments were compared based on the relative order of labels of different dog chromosomes syntenic to the cat’s genome.

The resulting synteny blocks cover 99% of the cat’s genome, while 81.7% of that agrees with the assignment of homologous chromosomes obtained by the chromosomal painting approach [20] (Fig. 1); halSynteny produced results different from the chromosomal painting results in the red regions of the cat chromosomes A1, B1, and C1. These regions were labeled as homologous to dog chromosome 28 with chromosome painting, while halSynteny revealed homology with chromosome 25. These regions comprise 3.5% of the constructed synteny.

Such discrepancies can stem from the different nature of the 2 approaches. While genome alignment depends on the accuracy of inferred genomic sequence, chromosomal painting results depend on the DNA composition and environment of a genomic region. Although chromosomal painting provides an efficient technique to discover large-scale similarity of continuous homology, it tends to misclassify small insertions and small translocations [21]. Also, it was reported that in cases of complex rearrangements chromosomal painting can be laborious and requires confirmation [22].

Comparison of gene-level orthology performed between the cat chromosomes A1, B1, and C1 and dog chromosomes 28 and 25 [23] using OMA Browser [24,25] support the conclusions of inference derived by halSynteny.

We also performed an evaluation of halSynteny performance in comparison with the SatsumaSynteny2 software [5, 26] because it is a modern method for synteny reconstruction based on inference directly from the genomic alignments (in contrast to anchor-based tools). A comparison was performed on the basis of the described protocol [14] for 2 datasets of genomes of nematodes: *Caenorhabditis elegans* (PRJNA13758) and *Caenorhabditisbriggsae* (PRJNA10731), *Strongyloides ratti*(PRJEB125) and *Strongyloides stercoralis* (PRJEB528). The time required for construction of the whole-genome alignments is not counted as part of halSynteny performance because such an alignment is needed for a realistic comparative genomic project separately. Such an alignment allows for investigation of sequence orthology, mapping of genomic markers among genomes, and other independent tasks. Finally it allows for better understanding of produced synteny blocks by uploading it into the UCSC Genome Browser [27,28].

Results are presented in Table 1. The results of SatsumaSynteny2 in terms of genome coverage are similar to the ones reported by the benchmark study [14] for the older version of the tool SatsumaSynteny [5]. It is possible to account for specific assembly qualities, such as the diverse size of assembly fragments, by adjusting the resolution parameters of halSynteny, which may result in an increase of genome coverage.

**Table 1:** Comparison of run time and genome coverage of resulting synteny blocks between SatsumaSynteny2 and halSynteny

Genome	Assembly N50 (Mb)	Genome coverage (%)		Time required, min	
		SatsumaSynteny2	halSynteny	SatsumaSynteny2	halSynteny
*Strongyloides ratti*	11.7	55.6	72.5	1,232	18 (+496)
*Strongyloides stercoralis*	0.4	56.6	55.5		
*Caenorhabditis elegans*	17.5	20.0	92.5	547	74 (+496)
*Caenorhabditisbriggsae*	108.4	18.7	88.3		

Comparison of run time and genome coverage of resulting synteny blocks between SatsumaSynteny2 and halSynteny. The former was run with extra parameter -threads 10. The latter was run with resolution parameters –maxAnchorDistance 1000000 (1 Mb), –minBlockSize 100000 (100 kb) for *S. ratti*/*S. stercoralis*, –maxAnchorDistance 1000000 (1Mb), –minBlockSize 1000000 (1 Mb) for *C. elegans*/*C. briggsae*. As a preliminary step for application of halSynteny, the whole-genome alignment among all 4 genomes was constructed using Progressive Cactus software, which took 496 minutes. The assemblies of *C. elegans* and *C. briggsae*are of chromosomal level, while there are scaffold-level assemblies for *S. ratti*and *S. stercoralis*.

## Discussion

Given an alignment of 2 genomes, information about their alignment with a third genome does not affect synteny between the original pair of genomes. Thus our approach can be scaled to the problem of multiple genome comparison without loss of precision. As a use case, given 3 genomes *G*_1_, *G*_2_, *G*_3_, where *G*_1_ is a reference genome, *G*_2_ is a genome of interest, and *G*_3_ is an outgroup genome, we can build synteny blocks between pairs of <*G*_1_, *G*_2_> and <*G*_1_, *G*_3_> and assign evolutionary breakages of lineages of genomes *G*_1_ and *G*_2_ using *G*_3_ as an outgroup.

halSynteny implements an algorithm for producing synteny blocks from genome alignment designed to process binary HAL files as input. The DAG-based method DAGchainer [4] was previously implemented for constructing synteny from the BLAST [29] alignments of gene annotations. It operates with homologous gene pairs found within complete genome sequences, combining them into chains of syntenic genes. The alignment-based method SatsumaSynteny2 takes pairs of genome sequences as input and implements a dynamic programming algorithm for chaining the pairwise alignment blocks. Here we first apply the DAG-based approach to whole-genome alignments. We define synteny for a pair of genomes, aiming for more accurate results obtained from multiple genome alignment. In comparison with the other modern alignment-based software, halSynteny allows for obtaining high-coverage results that follow from the definition of synteny. When the performance of halSynteny is compared to that of alignment-based software, halSynteny produces much higher genome coverage, which agrees with the properties of the dataset. These results are closer to the results of anchor-based tools reported in the benchmark study [14], while halSynteny does not require an intermediate genome annotation step. halSynteny can be installed as part of the halTools software essential for HAL file processing and can be a useful tool for analyzing whole-genome alignment data.

## Availability of Supporting Source Code and Requirements

An archival copy of the code and other supporting data is available via the *GigaScience* database, GigaDB [23].

Project name: halSynteny

Project home page: https://github.com/ComparativeGenomicsToolkit/hal

Operating system(s): Linux

Programming language: C++11

Other requirements: HAL API

License: MIT

RRID:SCR_018127

biotoolsID: biotools:halSynteny https://bio.tools/halSynteny

## Additional Files

**Figure 1:** DAG fragments corresponding to the comparison of *S. ratti* and *S. stercoralis*

## Abbreviations

API: Application Programming Interface; BLAST: Basic Local Alignment Search Tool; DAG: directed acyclic graph; kb: kilobase pairs; Mb: megabase pairs; NHGRI: National Human Genome Research Institute; RBH: reciprocal best hits; UCSC: University of California Santa Cruz.

## Funding

This publication was supported by a Subagreement from European Molecular Biology Laboratory with funds provided by Agreement No. 2U41HG007234-05 from National Institutes of Health, NIHGR. It was also supported by the NIHGR of the National Institutes of Health under Award No. R01HG008742. Its content is solely the responsibility of the authors and does not necessarily represent the official views of the National Institutes of Health, NIHGR, or European Molecular Biology Laboratory. K.K. was supported by RFBR grant, project No. 20-34-70055. K.K. and S.O. were supported, in part, by a Russian Science Foundation grant (project No. 17-14-01138) and by St. Petersburg State University (Genome Russia Grant No. 1.52.1647.2016).

## Competing Interests

The authors declare that they have no competing interests.

## Authors' Contributions

Method development: K.K., M.D., J.A.; Implementation and testing: K.K., M.D., J.A.; Data preparation: K.K.; Supervision: M.D., B.P., S.O.; Definition of research project: K.K.; Writing – review and editing: K.K., M.D., A.D., B.P., S.O.

## Supplementary Material

giaa047_GIGA-D-19-00419_Original_Submission

giaa047_GIGA-D-19-00419_Revision_1

giaa047_GIGA-D-19-00419_Revision_2

giaa047_Response_to_Reviewer_Comments_Original_Submission

giaa047_Response_to_Reviewer_Comments_Revision_1

giaa047_Reviewer_1_Report_Original_Submission Christophe Dessimoz -- 1/9/2020 Reviewed

giaa047_Reviewer_1_Report_Revision_1 Christophe Dessimoz -- 4/4/2020 Reviewed

giaa047_Reviewer_2_Report_Original_Submission Michael Hiller -- 1/11/2020 Reviewed

giaa047_Reviewer_2_Report_Revision_1 Michael Hiller -- 3/19/2020 Reviewed

giaa047_Supplemental_File
