# Supplementary material for: halSynteny: a fast, easy-to-use conserved synteny block construction method for multiple whole-genome alignments
Source: Gigascience. 2020 May 28;9(6):giaa047. doi: 10.1093/gigascience/giaa047 (PMC7254927; doi:10.1093/gigascience/giaa047)

GigaScience

halSynteny: a fast, easy-to-use conserved synteny block construction method for multiple whole-genome alignments

--Manuscript Draft--

**Manuscript Number:**	GIGA-D-19-00419
**Full Title:**	halSynteny: a fast, easy-to-use conserved synteny block construction method for multiple whole-genome alignments
**Article Type:**	Technical Note
**Funding Information:**	National Institute of Health (USA) (2U41HG007234-05) — Dr Stephen O'Brien<br>National Human Genome Research Institute (US) (R01HG008742) — Dr Stephen O'Brien<br>Russian Science Foundation (17-14-01138) — Ms Ksenia Krasheninnikova<br>St.Petersburg State University (1.52.1647.2016) — Dr Stephen O'Brien
**Abstract:**	Large-scale sequencing projects provide high-quality full genome data that can be used for reconstruction of chromosomal exchanges and rearrangements that disrupt conserved syntenic blocks. The highest resolution of cross-species homology can be obtained based on whole-genome, reference-free alignments. Very large multiple alignments of full-genome sequence stored in a binary format demand an accurate and efficient computational approach for synteny blocks production.<br>halSynteny performs efficient processing of pairwise alignment blocks for any pair of genomes in the alignment. The tool is part of the HAL comparative genomics suite and is targeted to build synteny blocks for multi-hundred way, reference-free vertebrate alignments built with the Cactus system.<br>halSynteny enables an accurate and rapid identification of synteny in multiple full-genome alignments. The method is implemented in C++11 as a component of the halTools software and released under MIT license. The package is available at https://github.com/ComparativeGenomicsToolkit/hal/.
**Corresponding Author:**	Ksenia Krasheninnikova<br><br>RUSSIAN FEDERATION
**Corresponding Author Secondary Information:**	
**Corresponding Author's Institution:**	
**Corresponding Author's Secondary Institution:**	
**First Author:**	Ksenia Krasheninnikova
**First Author Secondary Information:**	
**Order of Authors:**	Ksenia Krasheninnikova
	Mark Diekhans
	Joel Armstrong
	Alexei Dievskii
	Benedict Paten
	Stephen O'Brien
**Order of Authors Secondary Information:**	
**Additional Information:**	
**Question**	**Response**

Powered by Editorial Manager® and ProduXion Manager® from Aries Systems Corporation

Are you submitting this manuscript to a special series or article collection?	No
**Experimental design and statistics**<br><br>Full details of the experimental design and statistical methods used should be given in the Methods section, as detailed in our Minimum Standards Reporting Checklist. Information essential to interpreting the data presented should be made available in the figure legends.<br><br>Have you included all the information requested in your manuscript?	Yes
**Resources**<br><br>A description of all resources used, including antibodies, cell lines, animals and software tools, with enough information to allow them to be uniquely identified, should be included in the Methods section. Authors are strongly encouraged to cite Research Resource Identifiers (RRIDs) for antibodies, model organisms and tools, where possible.<br><br>Have you included the information requested as detailed in our Minimum Standards Reporting Checklist?	Yes
**Availability of data and materials**<br><br>All datasets and code on which the conclusions of the paper rely must be either included in your submission or deposited in publicly available repositories (where available and ethically appropriate), referencing such data using a unique identifier in the references and in the "Availability of Data and Materials" section of your manuscript.<br><br>Have you have met the above requirement as detailed in our Minimum	Yes

Powered by Editorial Manager® and ProduXion Manager® from Aries Systems Corporation

Standards Reporting Checklist?	

Powered by Editorial Manager® and ProduXion Manager® from Aries Systems Corporation

Main document
Click here to view linked References
Click here to access/download;Manuscript;GigScience-halSynteny.pdf

GigaScience, 2020, 1–4

doi: xx.xxxx/xxxx
Manuscript in Preparation
Paper

PAPER

halSynteny: a fast, easy-to-use conserved synteny block construction method for multiple whole-genome alignments

Ksenia Krasheninnikova[1,*], Mark Diekhans[2], Joel Armstrong[2], Aleksei Dievskii , Benedict Paten[2] and Stephen O'Brien[2,3,4]

[1]Computer Technologies Laboratory, ITMO University, St. Petersburg, Russian Federation and [2]UC Santa Cruz Genomics Institute, Santa Cruz, USA and [3]Theodosius Dobzhansky Center for Genome Bioinformatics St. Petersburg State University St. Petersburg, Russia 199004 and [4]Guy Harvey Oceanographic Center Halmos College of Natural Sciences and Oceanography Nova Southeastern University 8000 North Ocean Drive Ft Lauderdale, Florida 33004 USA

* kvkrasheninnikova@itmo.ru

Abstract

Background Large-scale sequencing projects provide high-quality full genome data that can be used for reconstruction of chromosomal exchanges and rearrangements that disrupt conserved syntenic blocks. The highest resolution of cross-species homology can be obtained based on whole-genome, reference-free alignments. Very large multiple alignments of full-genome sequence stored in a binary format demand an accurate and efficient computational approach for synteny blocks production.
Findings halSynteny performs efficient processing of pairwise alignment blocks for any pair of genomes in the alignment. The tool is part of the HAL comparative genomics suite and is targeted to build synteny blocks for multi-hundred way, reference-free vertebrate alignments built with the Cactus system.
Conclusions halSynteny enables an accurate and rapid identification of synteny in multiple full-genome alignments. The method is implemented in C++11 as a component of the halTools software and released under MIT license. The package is available at https://github.com/ComparativeGenomicsToolkit/hal/.

Key words: Synteny Blocks; Genome Alignments; Comparative Genomics; HAL format

Introduction

Conserved synteny blocks provide a conceptual framework for the analysis of interspecies homology. Originally, the notion of synteny stems from the area of cell genetics where it was defined as the co-location of two or more homologous genes on the same chromosome [1]. This term has been adopted by the comparative genomics field as contiguously aligned regions that preserve order and orientation of the alignment while allowing for micro-rearrangements within the syntenic region [2]. These genomics approaches introduce some quantitative properties of blocks, such as the size of blocks and resolution of synteny in bases.

There are a number of existing tools designed for finding synteny blocks [2, 3, 4, 5, 6, 7, 8, 9, 10]. These tools all require various data formats, which must be derived from the alignment, such as a predefined set of homologous genomic markers, or genome alignment blocks, each being a sequence of aligned bases that is contiguous in each of the genomes represented by the block. With halSynteny, the alignment is the

Compiled on: December 4, 2019.
Draft manuscript prepared by the author.

only required input.

With the increased availability of large scale computing facilities, multiple vertebrate whole-genome alignment is now tractable. Multi-species genome alignments are a useful tool for analysis of species homology in large-scale comparative genomic projects [11, 12]. One of the state-of-the-art tools [13, 14, 15] is Progressive Cactus [16, 17] which produces reference-free all-to-all genome alignments.

By producing a single, reference-free multiple alignment, Cactus allows synteny block reconstruction between any two genomes without reference bias, directly from the HAL representation. Here we present halSynteny tool that implements a directed acyclic graph (DAG)-based algorithm for identification of synteny blocks directly from HAL alignment and reporting synteny blocks in PSL format [18].

Methods

Algorithm. The algorithm operates on a pair of selected genome assemblies in the HAL multiple alignment. A synteny block is a sequence of local alignments that in each of genomes maintain the following properties: (a) are on one chromosome, (b) do not overlap, (c) are on the same strand, (d) have chromosome sequence coordinates that are either monotonically increasing (for positive strand) or decreasing for negative strand [2]. The set of synteny blocks over a pair of genomes is parameterized by the lower bound of minimal block length b_{min} and maximal distance d_{max} between two sequential anchoring alignment blocks. The pair (b_{min}, d_{max}) can be regarded as a resolution of the synteny block.

Each gapless alignment block between the pair of genomes is represented with the start and end positions on the chromosomes, along with the strand. Due to the modeling of duplications, different alignment blocks can overlap in coordinates in either genome.

The set of graph vertices V is formed by gapless alignment blocks. Vertex v_j is defined syntenic to v_i if each genome maintains the same order and orientation, their corresponding genomic coordinates do not overlap, and the genomic distance in either genome between v_i and v_j , d_{ij}, doesn't exceed the maximal distance dmax defined by the synteny resolution. The set of graph edges E is formed by all pairs (v_i, v_j) such that v_j is syntenic to v_i. This results in the set of DAG subgraphs corresponding to synteny regions in the graph of alignment blocks. In order to build a set of as many continuous synteny blocks as possible covering as much of both genome sequences as possible, we build the graph $G =< V, E >$ and apply the following algorithm:

i. Initialize weight labels of vertices and edges:

- Initialize the weight of each vertex w_{v_i} as the absolute value of the difference between start and end coordinates in target of query genomes, which is called size of the corresponding alignment block.
- The weight w_i of each edge coming into a vertex v_i is defined as the initial weight of the vertex v_i.

ii. Traverse the vertices in topological order.

- For each vertex, v_i consider all the edges (v_i, v_j) and for all incident vertices v_j calculate the candidate weight update defined as the weight of an edge coming into v_j plus the weight of the preceding vertex v_i.
- If the candidate weight is greater than the current weight of v_j replace the weight of v_i with this value.
- If w_i was updated, store the parent vertex v_i for backtracking.

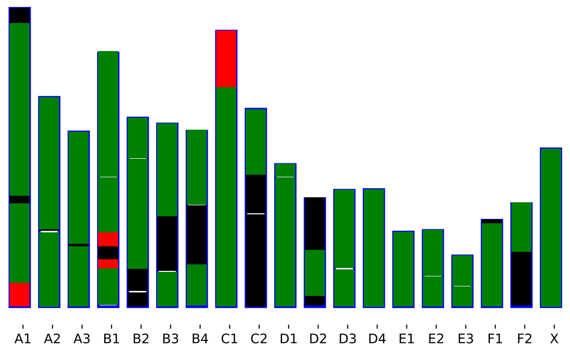

Figure 1. Comparison of produced synteny blocks between halSynteny and results obtained by chromosomal painting [19] of domestic cat chromosomes with thoses of domestic dog. Blue lines depict the borders of chromosomes. Green segments indicate regions where both methods have identified the same homologous regions in the dog genome (80.9% of cat genome). Red indicates regions where different homologous dog regions are identified (3.4%). Black stretches indicate regions not covered by chromosome painting where halSynteny has produced synteny blocks (14.6%). The white regions correspond to segments with no halSynteny blocks that may be covered partly by chromosome painting (0.01%). Since the chromosomal painting approach is not bound to any assembly and does not produce any genomic coordinates, two assignments were compared based on the relative order of labels of different dog chromosomes syntenic to the cat's genome.

iii. Find the vertex with maximal weight and trace back using stored previous vertices.

iv. If the path built from vertices obtained at step 3 is at least as long as the predefined minimal block length (defined by resolution), then remove them from the vertex set and store this path as a synteny block, else stop execution. If V is not empty then go to step iii.

As a result, we construct a set of possibly overlapping in genomic coordinates paths, so that each path covers as much of each genome as possible.

Evaluation of results. In order to assess the accuracy of this algorithm, we constructed synteny blocks between the domestic cat (FelCat 8.0) and domestic dog (CanFam 3.1) genomes based on Progressive Cactus alignment of these genomes together with the human genome (GRCh38) as an outgroup.

Resulting synteny blocks cover 99% of the cat's genome, while 81.7% of that agrees with the assignment of homologous chromosomes obtained by the chromosomal painting approach [19] (Figure 1); halSynteny produced results different from the chromosomal painting results in the purple regions of the cat's chromosomes A1, B1, and C1. These regions were labeled as homologous to dog's chromosome 28 with chromosome painting, while halSynteny revealed homology with chromosome 25. These regions comprise 3.5% of the constructed synteny.

We also performed an evaluation of halSynteny performance in comparison to the SatsumaSynteny2 software [4] (https://github.com/bioinfologics/satsuma2), as it is a modern method for synteny reconstruction based on inference directly from the genomic alignments (in contrast to anchor-based tools). A comparison was performed based on the described protocol [13] for two datasets of genomes of nematodes: C. elegans (PRJNA13758) and C. briggsae (PRJNA10731), S. ratti (PRJEB125) and S. stercoralis (PRJEB528). The time required for construction of the whole genome alignments is not counted as part of halSynteny performance, because such an alignment is needed for a realistic comparative genomic project separately. Such an alignment allows for investigation of sequence orthol-

Table 1. Comparison of run time and genome coverage of resulting synteny blocks between SatsumaSynteny2 and halSynteny

Genome	Assembly N50 (Mbp)	Genome coverage (%)		Time required, min	
		SatsumaSynteny2	halSynteny	SatsumaSynteny2	halSynteny
S.ratti	11.7	55.6	72.5	1232	18
S. stercoralis	0.4	56.6	55.5		
C.elegans	17.5	20.0	92.5	547	74
C.briggsae	108.4	18.7	88.3		

Comparison of run time and genome coverage of resulting synteny blocks between SatsumaSynteny2 and halSynteny. The former was run with extra parameter -threads 10. The latter was run with resolution parameters –maxAnchorDistance 1000000 (1Mb), –minBlockSize 100000 (100Kb) for S.ratti/S.stercoralis, –maxAnchorDistance 1000000 (1Mb), –minBlockSize 1000000 (1Mb) for C.elegans/C.briggsae. As a preliminary step for application of halSynteny, the whole-genome alignment among all four genomes was constructed using Progressive Cactus software, which took 496 minutes.

ogy, mapping of genomic markers among genomes, and other independent tasks.

Finally it allows for better understanding of produced synteny blocks by uploading it into the UCSC Genome Browser [20, 21].

Results are presented in Table 1. The results of SatsumaSynteny2 in terms of genome coverage are similar to the ones reported by the benchmark study [13] for the older version of the tool SatsumaSynteny [4]. It's possible to account for specific assembly qualities, such as the diverse size of assembly fragments, by adjusting resolution parameters of halSynteny, which may result in an increase of genome coverage.

Discussion

Given an alignment of two genomes, information about their alignment with a third genome does not affect synteny between the original pair of genomes. Thus our approach can be scaled to the problem of multiple genomes comparison without loss of precision. As a use case, given three genomes G_1,G_2,G_3, where G_1 is a reference genome, G_2 is a genome of interest, G_3 is an outgroup genome, we can build synteny blocks between pairs of $< G_1, G_2 >$ and $< G_1, G_3 >$ and assign evolutionary breakages of lineages of genomes G_1 and G_2 using G_3 as an outgroup.

halSynteny implements an algorithm for producing synteny blocks from genome alignment designed to process binary HAL files as input. The DAG-based method DAGchainer [3] was previously implemented for constructing synteny from the blast [22] alignments of gene annotations. It operates with homologous gene pairs found within complete genome sequences combining them into chains of syntenic genes. An alignment-based method SatsumaSynteny2 takes pairs of genome sequences as input and implements a dynamic programming algorithm for chaining the pairwise alignment blocks. Here we first apply the DAG-based approach to the whole-genome alignments. We define synteny for a pair of genomes aiming for more accurate results obtained from multiple genome alignment. Comparing to the other modern alignment-based software halSynteny allows for obtaining high-coverage results which follow from the definition of synteny. When comparing the performance of halSynteny to alignment-based software halSynteny produces much higher genome coverage, which agrees with the properties of the dataset. These results are closer to the results of anchor-based tools reported in the benchmark study [13], while halSynteny does not require intermediate genome annotation step. halSynteny can be installed as part of the halTools software essential for HAL-file processing and can be a useful tool when analyzing the whole-genome alignment data.

Funding

This publication was supported by a Subagreement from European Molecular Biology Laboratory with funds provided by Agreement No. 2U41HG007234-05 from National Institute of Health, NIGHR. It was also supported by the National Human Genome Research Institute of the National Institutes of Health under Award Number R01HG008742. Its content is solely the responsibility of the authors and do not necessarily represent the official views of National Institute of Health, NHGRI or European Molecular Biology Laboratory. Ksenia Krasheninnikova and Stephen O'Brien were supported, in part, by the Russian Science Foundation grant (project No. 17-14-01138) and by St. Petersburg State University (Genome Russia Grant no. 1.52.1647.2016).

Competing Interests

The author(s) declare that they have no competing interests.

Author's Contributions

Method development: K.K., M.D., J.A.; Implementation and testing: K.K., M.D., J.A.; Data preparation: K.K.; Supervision: M.D, B.P., S.J.B.; Definition of research project: K.K; Writing – review editing: K.K., M.D., A.D, B.P, S.J.B

References

1. Hickey G, Paten B, Zerbino D, Haussler D. HAL: A hierarchical format for storing and analyzing multiple genome alignments. Bioinformatics 2013;.
2. Pevzner P, Tesler G. Genome rearrangements in mammalian evolution: lessons from human and mouse genomes. Genome Research 2003;.
3. Haas BJ, Delcher AL, R WJ, Salzberg SL. DAGchainer: A tool for mining segmental genome duplications and synteny. Bioinformatics 2004;.
4. Grabherr MG, Russell P, Meyer M, Mauceli E, Alföldi J, di Palma F, et al. Genome-wide synteny through highly sensitive sequence alignment: Satsuma. Bioinformatics 2010;.
5. Wang Y, Tang H, Debarry JD, Tan X, Li J, Wang X, et al. MCScanX: A toolkit for detection and evolutionary analysis of gene synteny and collinearity. Nucleic Acid Research 2012;.
6. Proost S, Fostier J, De Witte D, Dhoedt B, Demeester P, Van De Peer Y, et al. i-ADHoRe 3.0-fast and sensitive detection of genomic homology in extremely large data sets. Nucleic Acid Research 2012;.
7. Drillon G, Carbone A, Fischer G. SynChro: A fast and easy

tool to reconstruct and visualize synteny blocks along eukaryotic chromosomes. PLoS One 2014;.
8. Pham SK, Pevzner PA. DRIMM-Synteny: Decomposing genomes into evolutionary conserved segments. Bioinformatics 2014;.
9. Soderlund C, Bomhoff M, Nelson WM. SyMAP v3.4: A turnkey synteny system with application to plant genomes. Nucleic Acid Research 2011;.
10. Rödelsperger C, Dieterich C. CYNTENATOR: Progressive gene order alignment of 17 vertebrate genomes. PLoS One 2010;.
11. Lilue J, Doran AG, Fiddes IT, Abrudan M, Armstrong J, Bennett R, et al. Sixteen diverse laboratory mouse reference genomes define strain-specific haplotypes and novel functional loci. Nature Genetics 2018;.
12. Zhang G, Li C, Li Q, Li B, Larkin DM, Lee C, et al. Comparative genomics reveals insights into avian genome evolution and adaptation. Science 2014;.
13. Liu D, Hunt M, Tsai IJ. Inferring synteny between genome assemblies: A systematic evaluation. BMC Bioinformatics 2018;.
14. Dobrynin P, Liu S, Tamazian G, Xiong Z, Yurchenko AA, Krasheninnikova K, et al. Genomic legacy of the African cheetah, Acinonyx jubatus. Genome Biology 2015;.
15. Choo SW, Rayko M, Tan TK, Hari R, Komissarov A, Wee WY, et al. Pangolin genomes and the evolution of mammalian scales and immunity. Genome Research 2016;.
16. Paten B, Earl D, Nguye nN, Diekhans M, Zerbino D, Haussler D. Cactus: Algorithms for genome multiple sequence alignment. Genome Research 2011;.
17. Paten B, Diekhans M, Earl D, John JS, Ma J, Suh B, et al. Cactus graphs for genome comparisons. Computer Science 2010;.
18. Schlenoff C, Gruninger M. The Process Specification Language (PSL) Overview and Version 1.0 Specification. Technical Reports 2004;.
19. Yang F, Graphodatsky A, O'Brien P, Colabella A, Solanky N, Squire M, et al. The Process Specification Language (PSL) Overview and Version 1.0 Specification. Chromosome Research 2000;.
20. Kent WJ, Sugnet CW, Furey TS, Roskin KM, Pringle TH, Zahler AM, et al. The human genome browser at UCSC. Genome Research 2002;.
21. Raney BJ, Dreszer TR, Barber GP, Clawson H, Fujita PA, Wang T, et al. Track data hubs enable visualization of user-defined genome-wide annotations on the UCSC Genome Browser. Bioinformatics 2014;.
22. Altschul SF, Gish W, Miller W, Myers EW, Lipman DJ. Basic local alignment search tool. Journal of Molecular Biology 1990;.

Figure 1

Click here to access/download;Figure;figure1_cat_dog_red-

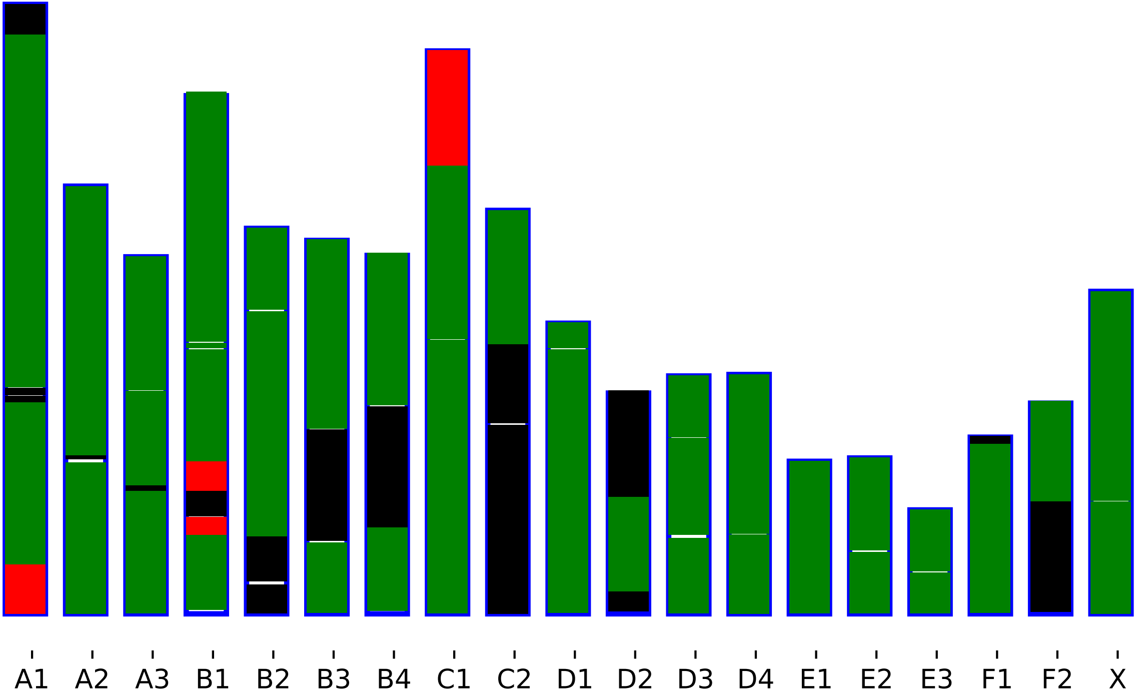

Supplement: giaa047_GIGA-D-19-00419_Original_Submission [file giaa047_giga-d-19-00419_original_submission.pdf]
