# Supplementary material for: halSynteny: a fast, easy-to-use conserved synteny block construction method for multiple whole-genome alignments
Source: Gigascience. 2020 May 28;9(6):giaa047. doi: 10.1093/gigascience/giaa047 (PMC7254927; doi:10.1093/gigascience/giaa047)

GigaScience

halSynteny: a fast, easy-to-use conserved synteny block construction method for multiple whole-genome alignments

--Manuscript Draft--

**Manuscript Number:**	GIGA-D-19-00419R1
**Full Title:**	halSynteny: a fast, easy-to-use conserved synteny block construction method for multiple whole-genome alignments
**Article Type:**	Technical Note

Funding Information:

Funder	Recipient
National Institute of Health (USA) (2U41HG007234-05)	Dr Stephen O'Brien
National Human Genome Research Institute (US) (R01HG008742)	Dr Stephen O'Brien
Российский Фонд Фундаментальных Исследований (РФФИ) (20-34-70055)	Ms Ksenia Krasheninnikova
Russian Science Foundation (17-14-01138)	Ms Ksenia Krasheninnikova
St.Petersburg State University (1.52.1647.2016)	Dr Stephen O'Brien

Abstract:

Large-scale sequencing projects provide high-quality full genome data that can be used for reconstruction of chromosomal exchanges and rearrangements that disrupt conserved syntenic blocks. The highest resolution of cross-species homology can be obtained based on whole-genome, reference-free alignments. Very large multiple alignments of full-genome sequence stored in a binary format demand an accurate and efficient computational approach for synteny blocks production.
halSynteny performs efficient processing of pairwise alignment blocks for any pair of genomes in the alignment. The tool is part of the HAL comparative genomics suite and is targeted to build synteny blocks for multi-hundred way, reference-free vertebrate alignments built with the Cactus system.

halSynteny enables an accurate and rapid identification of synteny in multiple full-genome alignments. The method is implemented in C++11 as a component of the halTools software and released under MIT license. The package is available at https://github.com/ComparativeGenomicsToolkit/hal/.

**Corresponding Author:**	Ksenia Krasheninnikova<br><br>RUSSIAN FEDERATION
**Corresponding Author Secondary Information:**	
**Corresponding Author's Institution:**	
**Corresponding Author's Secondary Institution:**	
**First Author:**	Ksenia Krasheninnikova
**First Author Secondary Information:**	
**Order of Authors:**	Ksenia Krasheninnikova
	Mark Diekhans
	Joel Armstrong
	Alexei Dievskii
	Benedict Paten
	Stephen O'Brien

Powered by Editorial Manager® and ProduXion Manager® from Aries Systems Corporation

**Order of Authors Secondary Information:**	
**Response to Reviewers:**	Dear Editor and Reviewers,<br>We do appreciate the reviewers' comments and their helpful suggestions for improving our manuscript. Based on these suggestions we included some elaborations into the text. We believe these changes have improved the quality and value of our work. A point-by-point response to the comments can be found below:<br>Reviewer #1:<br>This technical note describes a tool to compute synteny blocks from multiple genome alignments in HAL format, such as those produced by the tool Progressive Cactus. The paper is a good fit to the editorial requirements of a technical note. The problem tackled is relevant. The algorithm is well described and makes sense. The code is available under an open access license.<br>I only have a couple of minor points that feel ought to be addressed before this paper can be accepted.<br>Minor points<br>- The documentation is very sparse. At the minimum, describe all the possible arguments.<br><br>Thank you. The documentation at github has been updated.<br><br>- Introduction: "There are a number of existing tools designed for finding synteny blocks [2, 3, 4, 5, 6, 7, 8, 9, 10]". It would be more reader-friendly if the authors provided a bit more context on these various citations.<br><br>We extended the description of the available software in the area of synteny blocks construction in the text.<br><br>- "Due to the modeling of duplications, different alignment blocks can overlap in coordinates in either genome.". Provide more explanations about how duplications are handled, as this is central to this problem.<br><br>Each gapless alignment block between the pair of genomes is represented with the start and end positions on the chromosomes, along with the strand. Duplications are expressed as overlapping alignment blocks. Presume there are alignment hits in genome A that can be ordered by genomic coordinates as p1 ···pi,···pn and in the genome B as u1 ···ui,ui+1,···un+1, and there are alignments present among segments pl and ul for l ∊ 1 · · · i pk and uk+1 for k ∊ i · · · n. Then the synteny blocks between the genomes A and B can overlap and contain the following pairs of segments: (pi, ui) for l ∊ 1 · · · i and (pk, uk+1) for k ∊ i···n.<br>We included this explanation into the text of the manuscript. Also, please, see Supplementary Figure 1 for illustration of handling duplications.<br><br>- Differences between halSynteny and chromosomal painting in red segments A1, B1, and C1. It should be possible to resolve the discrepancy. I had a look at the synteny dot plot in the OMA database, where orthology is inferred at the level of individual genes, and it appears that all three are syntenic with dog chromosome 25 and not dog chromosome 28.<br>orthologous segments here:<br>https://omabrowser.org/oma/dotplot/FELCA/CANLF/A1/25/<br>https://omabrowser.org/oma/dotplot/FELCA/CANLF/B1/25/<br>https://omabrowser.org/oma/dotplot/FELCA/CANLF/C1/25/<br>No orthology here:<br>https://omabrowser.org/oma/dotplot/FELCA/CANLF/A1/28/<br>https://omabrowser.org/oma/dotplot/FELCA/CANLF/B1/28/<br>https://omabrowser.org/oma/dotplot/FELCA/CANLF/C1/28/<br><br>We thank the Reviewer for this valuable addition to the question of contradictory analysis of synteny for the dog chromosomes 25 and 28. This valuable evidence supports the conclusions based on the whole-genome alignments described in the manuscript.<br><br>Discretionary:<br>- The algorithm is provided without proof on correctness, or analysis on asymptotic time and space costs. Perhaps worth including these if you have them or can do them

Powered by Editorial Manager® and ProduXion Manager® from Aries Systems Corporation

easily.

We thank the Reviewer for this valuable remark, as it's worth emphasizing that the algorithm is heuristical. In the course of the problem solution we desire to achieve two goals: the desired set of synteny blocks should contain as many con- tinuous synteny blocks as possible covering as much of both genome sequences as possible. Although we do not provide a strict mathematical proof of conditions which would always allow that both goals are achievable simultaneously, we describe the heuristic that strives to achieve them. The manuscript text is updated with more detailed definitions.
In the implementation of this algorithm available on github we store the graph as adjacency list which allows for asymptotic time and space complexity O(|V |+ |E |).

Typos
- Ref. 19 seems not to be the correct one for chromosomal painting. Thank you. It's fixed.
- Italicise species names in Table 1.

Fixed.

- On the GitHub README file, "Genom1" → "Genome1"

Thank you. Fixed.

Reviewer #2:
This manuscript describes a new tool, HalSynteny, that uses a dynamic programming algorithm to extract maximal pairwise syntenic regions from pairwise or multiple whole genome alignments. The tool is substantially faster than a previous method and extracts much larger syntenic regions. Thus, HalSynteny will likely have broad applicability.
I have only a few comments that I hope are helpful, and some questions.
1) The initial weight of vertex wvi is the length of the ungapped alignment block. I was wondering why not the alignment score (e.g. using a simple scoring scheme like +1 for a match as in Blat) is chosen. If there are two ungapped alignment blocks of equal size but one with a higher identity (thus alignment score), it would be preferable to include this block in the syntenic block.

We thank Professor Hiller for the reasonable suggestion. In fact, the goal was to find the largest continuous regions, while the identification of differences was not in scope. Here we rely on the alignment which has already established the most probable ordering.

2) In my printout, I can't really see the blue lines in figure 1. Also, the text writes about 'purple regions' but I think this is indicated as black in the figure. The blue lines refer to the contour of the schematic representation of chromosome on the Figure 1 in main text. We updated the text with regard to this concern.

We apologize for the misprint, since the 'purple regions' should be 'red regions'. This is corrected in the manuscript.

3) While I believe that HalSynteny is much more accurate than chromosome painting, also because it is based on actual sequence alignments, I was wonder- ing what could possibly explain the large difference between both methods for the cat chromosomes A1, B1, C1.

There are studies [1], [2] describing possibilities and limitations of the chromosomal painting approach. Although this approach provides an efficient technique to discover large-scale similarity of continuous homology, it tends to misclassify small insertions and small translocations [1]. Also, it was reported that in cases of complex rearrangements chromosomal painting can be laborious and requires confirmation [2]. Our results supported by the orthologous alignments kindly provided by the Reviewer 1 advocate that the identified blocks of chromosomes A1, B1, and C1 are syntenic to the dog chromosome 25.
Lastly, the synteny parameters (bmin, dmax) used were both bmin and dmax were set as 1Mb. This has been inserted into the text.

Powered by Editorial Manager® and ProduXion Manager® from Aries Systems Corporation

4) The authors could discuss in the text that HalSynteny is substantially faster than SatsumaSynteny2. I assume here that the runtime of SatsumaSynteny2 also does not include the time needed to produce the input genome alignment. Otherwise, this should be included in HalSynteny or excluded in SatsumaSynteny2 to make the runtime comparable.

Indeed, the alignment procedure requires most significant portion of time in both approaches to the synteny block construction. However in case of halSynteny, which uses alignment as input, the alignment procedure is self-sufficient and the produced output can be used independently for other analysis. SatsumaSynteny2 operates over fasta files as input and produces internal alignment which is not separable from results that's why the timing for SatsumaSynteny2 includes also alignment step. In this manuscript to benchmark halSynteny by construct- ing blocks between S.ratti and S.stercoralis, and separately for C.elegans and C.briggsae, we performed one alignment of all 4 species which took 496 minutes. After that running halSynteny over the alignment took 18 min and 74 min. We understand some uncertainty which arises from different approaches and added the alignment time in parentheses to the table along with its mentioning in the table's caption.

5) There are substantial differences in the coverage of syntenic blocks between both methods. Is this due to different synteny parameters? E.g. is SatsumaSynteny2 requiring much larger and more stringent syntenic blocks? If I understand the HalSynteny algorithm correctly, a syntenic block can 'skip over' a local inversion or local duplication without breaking the syntenic block. Maybe SatsumaSynteny2 does not possess this feature and stops at local rearrangements, which could explain these performance differences.

We used the comparison protocol described in [3]. The authors also observe that "Satsuma demonstrates fewer contiguation-dependent patterns as its detection of synteny relies on nucleotide alignments". This hypothesis may not be correct since our algorithm also employs nucleotide alignment but demonstrates higher coverage on the same data. Satsuma implements a completely different method based on the Fast Furier transform over the signal represented by nucleotide multiplication pattern. We can assume that the internal parameters limiting the enough bounds for the signal affect the coverage of synteny blocks.

Other suggestions: - I would merge the 'Finally it allows ..." sentence with the previous paragraph.

Thank you. Done.

- Discussion: While not the focus of the paper, I wonder whether a < G2,G3 > comparison would be necessary to detect breakages that occurred in the lineage leading to G2 (I think this is not detectable from a < G1, G2 > and < G1, G3 > comparison).

Usually an outgroup genome is selected for assigning breakpoints to G1 or G2, if we would like to consider breakages between G2 and G3 we would need to make a comparison < G2,G3 > as well as to use another genome G4 evolutionary distant enough from both as an outgroup.

- As a suggestion, a figure sketch illustrating the graph and a maximal syntenic block would be helpful. Maybe one could illustrate a case of skipping over a local inversion or duplication.

Thank you for this suggestion. Supplementary Figure 1 illustrates the process of synteny block construction. The alignment blocks for each query chromosome are numbered according to their appearance in the alignment traversal. Blocks 6152 and 6154 in the Supplemetary Figure 1b correspond to a genomic duplication. The algorithm described in the manuscript constructed a synteny block A from alignment blocks 6161,6157, 6152, 6149, etc. Depending on the threshold parameter regulating the minimal size of a synteny block alignment blocks 6121, 6150, 6154 could consititute another synteny block B which would overlap in genomic coordinates with

Powered by Editorial Manager® and ProduXion Manager® from Aries Systems Corporation

	A. If the genomic size of the region corresponding to blocks 6121, 6150, 6154 is less than the defined parameter, then the corresponding genomic regions won't be covered by synteny blocks.<br>Supplementary Figure 1 is obtained using Bandage software [4].<br><br>- Assembly N50 probably refers to scaffold N50?<br><br>Thank you for this remark. The assemblies of C.elegans and C.briggsae are of chromosomal level, while there are scaffold-level assemblies for S.ratti and S.stercoralis. This has been added to the main text.<br><br>- Michael Hiller<br><br>References<br>[1] C. Lee et al. "Limitations of Chromosome Classification by Multicolor Karyotyping". In: AJHG (2001).<br>[2] T. Ried et al. "Chromosome Painting: A Useful Art". In: Human Molecular Genetics (1998).<br>[3] D. Liu and I. J. Hunt M.and Tsai. "Inferring synteny between genome assemblies: A systematic evaluation". In: BMC Bioinformatics (2018).<br>[4] R.R. Wick et al. "Bandage: interactive visualisation of de novo genome assemblies". In: Bioinformatics (2015).
**Additional Information:**	
**Question**	**Response**
Are you submitting this manuscript to a special series or article collection?	No
**Experimental design and statistics**<br><br>Full details of the experimental design and statistical methods used should be given in the Methods section, as detailed in our Minimum Standards Reporting Checklist. Information essential to interpreting the data presented should be made available in the figure legends.<br><br>Have you included all the information requested in your manuscript?	Yes
**Resources**<br><br>A description of all resources used, including antibodies, cell lines, animals and software tools, with enough information to allow them to be uniquely identified, should be included in the Methods section. Authors are strongly encouraged to cite Research Resource Identifiers (RRIDs) for antibodies, model organisms and tools, where possible.	Yes

Powered by Editorial Manager® and ProduXion Manager® from Aries Systems Corporation

Have you included the information requested as detailed in our Minimum Standards Reporting Checklist?	
**Availability of data and materials**<br><br>All datasets and code on which the conclusions of the paper rely must be either included in your submission or deposited in publicly available repositories (where available and ethically appropriate), referencing such data using a unique identifier in the references and in the “Availability of Data and Materials” section of your manuscript.<br><br>Have you have met the above requirement as detailed in our Minimum Standards Reporting Checklist?	Yes

Main Text Updated
Click here to access/download;Manuscript;main_text_revision.pdf

OXFORD
(GIGA)^n SCIENCE

GigaScience, 2020, 1–4

doi: xx.xxxx/xxxx
Manuscript in Preparation
Paper

PAPER

halSynteny: a fast, easy-to-use conserved synteny block construction method for multiple whole-genome alignments

Ksenia Krasheninnikova[1,2*], Mark Diekhans[3], Joel Armstrong[3], Aleksei Dievskii , Benedict Paten[3] and Stephen O'Brien[1,4]

[1]Computer Technologies Laboratory, ITMO University, St. Petersburg, Russian Federation and [2]Wellcome Trust Sanger Institute, Wellcome Trust Genome Campus, Hinxton, CB10 1SA, UK and [3]UC Santa Cruz Genomics Institute, Santa Cruz, USA and [4]Guy Harvey Oceanographic Center Halmos College of Natural Sciences and Oceanography Nova Southeastern University 8000 North Ocean Drive Ft Lauderdale, Florida 33004 USA

[*]kvkrasheninnikova@itmo.ru

Abstract

Background Large-scale sequencing projects provide high-quality full genome data that can be used for reconstruction of chromosomal exchanges and rearrangements that disrupt conserved syntenic blocks. The highest resolution of cross-species homology can be obtained based on whole-genome, reference-free alignments. Very large multiple alignments of full-genome sequence stored in a binary format demand an accurate and efficient computational approach for synteny blocks production.
Findings halSynteny performs efficient processing of pairwise alignment blocks for any pair of genomes in the alignment. The tool is part of the HAL comparative genomics suite and is targeted to build synteny blocks for multi-hundred way, reference-free vertebrate alignments built with the Cactus system.
Conclusions halSynteny enables an accurate and rapid identification of synteny in multiple full-genome alignments. The method is implemented in C++11 as a component of the halTools software and released under MIT license. The package is available at https://github.com/ComparativeGenomicsToolkit/hal/.

Key words: Synteny Blocks; Genome Alignments; Comparative Genomics; HAL format

Introduction

Conserved synteny blocks provide a conceptual framework for the analysis of interspecies homology. Originally, the notion of synteny stems from the area of cell genetics where it was defined as the co-location of two or more homologous genes on the same chromosome [1]. This term has been adopted by the comparative genomics field as contiguously aligned regions that preserve order and orientation of the alignment while allowing for micro-rearrangements within the syntenic region [2]. These genomics approaches introduce some quantitative properties of blocks, such as the size of blocks and resolution of synteny in bases.

There are a number of existing tools designed for finding synteny blocks. GRIMM-Synteny [2] algorithm reconstructs an anchor graph from the predifined set of homologous hits shared by genomes, which can be local pairwise alignments or orthologous genes. Chains-and-Nets algorithm [3] introduces a novel *blastz* scoring scheme for identification of alignment anchors between two species. A chained alignment is built over

Compiled on: March 16, 2020.
Draft manuscript prepared by the author.

an ordered sequence of traditional pairwise nucleotide alignments, then the set of chains is processed into nets using the chains with the highest score. DAGchainer [4] implements a DAG-based approach over predefined pairs of gene anchors. Satsuma [5] describes application of the fast Fourier transform algorithm over the signal represented by nucleotide multiplication pattern. MCScanX [6] operates over the gene sets and applies a tuned scoring schemes in the dynamic programming algorithm over chains of pair-wise gene alignments. i-ADHoRe [7] introduces homology matrices to resolve homology among tandem replications of genes, further the Needleman-Wunsch algorithm is applied for detection of collinearity. SynChro [8] operates over Reciprocal Best Hists obtained from *blastp* alignments for reconstruction of the synteny blocks backbones. In comparison to GRIMM-Synteny, which allows local disruptions of synteny measured in genomic intervals, SynChro allows for unlimited number of non-RBH genes but preserves the required number of intermediate RBH genes between first and last gene in a synteny block. DRIMM-Synteny [9] algorithms implements application of A-Bruijn graphs over a set of predefined anchors. Analogously to GRIMM-Synteny it can be e.g. local alignments or pairs of similar genes. In contrast to GRIMM-Synteny it provides a resistance against unwanted synteny disruption when a search is performed over multiple genomes and homologous anchors may be absent in a small proportion of the genomes analyzed. The SyMAP [10] algorithm computes the raw hits between nucleotide sequences of a pair of genomes, which are then clustered and filtered using the optional gene annotation. CYNTENATOR [11] employs phylogenetic information and performs progressive alignment of the gene order among multiple genomes.

These tools all require various data formats, which must be derived from the alignment, such as a predefined set of homologous genomic markers, or genome alignment blocks, each being a sequence of aligned bases that is contiguous in each of the genomes represented by the block. Many of them also require a rigorous and reliable annotation of orthologous genes. With halSynteny, the alignment is the only required input.

With the increased availability of large scale computing facilities, multiple vertebrate whole-genome alignment is now tractable. Multi-species genome alignments are a useful tool for analysis of species homology in large-scale comparative genomic projects [12, 13]. One of the state-of-the-art tools [14, 15, 16] is Progressive Cactus [17, 18] which produces reference-free all-to-all genome alignments.

By producing a single, reference-free multiple alignment, Cactus allows synteny block reconstruction between any two genomes without reference bias, directly from the HAL representation. Here we present halSynteny tool that implements a directed acyclic graph (DAG)-based algorithm for identification of synteny blocks directly from HAL alignment and reporting synteny blocks in PSL format [19].

Methods

Algorithm. We describe an heuristical algorithm which operates on a pair of selected genome assemblies in the HAL multiple alignment. A synteny block is a sequence of local alignments that in each of genomes maintain the following properties: (a) are on one chromosome, (b) do not overlap, (c) are on the same strand, (d) have chromosome sequence coordinates that are either monotonically increasing (for positive strand) or decreasing for negative strand [2]. The set of synteny blocks over a pair of genomes is parameterized by the lower bound of minimal block length b_{min} and maximal distance d_{max} between two sequential anchoring alignment blocks. The pair (b_{min}, d_{max}) can be regarded as a resolution of the synteny block.

Each gapless alignment block between the pair of genomes is represented with the start and end positions on the chromosomes, along with the strand. Duplications are expressed as overlapping alignment blocks. Presume there are alignment hits in genome A that can be ordered by genomic coordinates as $p_1 \cdots p_i, \cdots p_n$ and in the genome B as $u_1 \cdots u_i, u_{i+1}, \cdots u_{n+1}$, and there are alignments present among segments p_l and u_l for $l \in 1 \cdots i$ p_k and u_{k+1} for $k \in i \cdots n$. Then the synteny blocks between these two genomes can overlap and contain the following pairs of segments: (p_i, u_i) for $l \in 1 \cdots i$ and (p_k, u_{k+1}) for $k \in i \cdots n$.

The set of graph vertices V is formed by alignment blocks. Vertex v_j is defined syntenic to v_i if each genome maintains the same order and orientation, their corresponding genomic coordinates do not overlap, and the genomic distance in either genome between v_i and v_j , d_{ij}, doesn't exceed the maximal distance dmax defined by the synteny resolution. The set of graph edges E is formed by all pairs (v_i, v_j) such that v_j is syntenic to v_i. This results in the set of DAG subgraphs corresponding to synteny regions in the graph of alignment blocks. The desirable set of synteny blocks would contain as many continuous synteny blocks as possible covering as much of both genome sequences as possible. To achieve this goal we build the graph $G =< V, E >$ and apply the following algorithm:

i. Initialize weight labels of vertices and edges:

- Initialize the weight of each vertex w_{v_i} as the absolute value of the difference between start and end coordinates in target of query genomes, which is called size of the corresponding alignment block.
- The weight w_i of each edge coming into a vertex v_i is defined as the initial weight of the vertex v_i.

ii. Traverse the vertices in topological order.

- For each vertex, v_i consider all the edges (v_i, v_j) and for all incident vertices v_j calculate the candidate weight update defined as the weight of an edge coming into v_j plus the weight of the preceding vertex v_i.
- If the candidate weight is greater than the current weight of v_j replace the weight of v_j with this value.
- If w_j was updated, store the parent vertex v_i for backtracking.

iii. Find the vertex with maximal weight and trace back using stored previous vertices.
iv. If the path built from vertices obtained at step 3 is at least as long as the predefined minimal block length (defined by resolution), then remove them from the vertex set and store this path as a synteny block, else stop execution. If V is not empty then go to step iii.

As a result, we construct a set of possibly overlapping in genomic coordinates paths, so that each path covers as much of each genome as possible.

Evaluation of results. In order to assess the accuracy of this algorithm, we constructed synteny blocks between the domestic cat (FelCat 8.0) and domestic dog (CanFam 3.1) genomes based on Progressive Cactus alignment of these genomes together with the human genome (GRCh38) as an outgroup.

Resulting synteny blocks cover 99% of the cat's genome, while 81.7% of that agrees with the assignment of homologous chromosomes obtained by the chromosomal painting approach [20] (Figure 1); halSynteny produced results different from the chromosomal painting results in the red regions of the cat's chromosomes A1, B1, and C1. These regions were labeled as homologous to dog's chromosome 28 with chromosome paint-

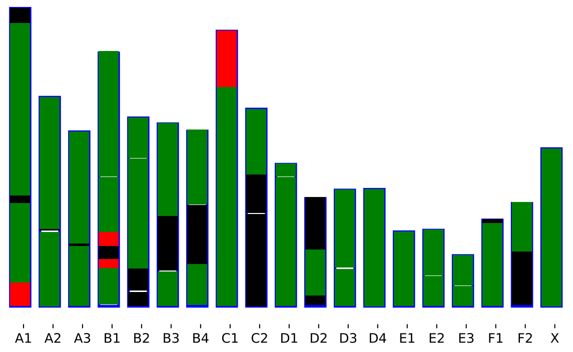

Figure 1. Comparison of produced synteny blocks between halSynteny with parameters –maxAnchorDistance 1000000 (1Mb), –minBlockSize 1000000 (1Mb) and results obtained by chromosomal painting [20] of domestic cat chromosomes with thoses of domestic dog. Blue contour depicts the borders of chromosomes. Green segments indicate regions where both methods have identified the same homologous regions in the dog genome (80.9% of cat genome). Red indicates regions where different homologous dog regions are identified (3.4%). Black stretches indicate regions not covered by chromosome painting where halSynteny has produced synteny blocks (14.6%). The white regions correspond to segments with no halSynteny blocks that may be covered partly by chromosome painting (0.01%). Since the chromosomal painting approach is not bound to any assembly and does not produce any genomic coordinates, two assignments were compared based on the relative order of labels of different dog chromosomes syntenic to the cat's genome.

ing, while halSynteny revealed homology with chromosome 25. These regions comprise 3.5% of the constructed synteny.

We also performed an evaluation of halSynteny performance in comparison to the SatsumaSynteny2 software [5] (https://github.com/bioinfologics/satsuma2), as it is a modern method for synteny reconstruction based on inference directly from the genomic alignments (in contrast to anchor-based tools). A comparison was performed based on the described protocol [14] for two datasets of genomes of nematodes: C. elegans (PRJNA13758) and C. briggsae (PRJNA10731), S. ratti (PRJEB125) and S. stercoralis (PRJEB528). The time required for construction of the whole genome alignments is not counted as part of halSynteny performance, because such an alignment is needed for a realistic comparative genomic project separately. Such an alignment allows for investigation of sequence orthology, mapping of genomic markers among genomes, and other independent tasks. Finally it allows for better understanding of produced synteny blocks by uploading it into the UCSC Genome Browser [21, 22].

Results are presented in Table 1. The results of SatsumaSynteny2 in terms of genome coverage are similar to the ones reported by the benchmark study [14] for the older version of the tool SatsumaSynteny [5]. It's possible to account for specific assembly qualities, such as the diverse size of assembly fragments, by adjusting resolution parameters of halSynteny, which may result in an increase of genome coverage.

Discussion

Given an alignment of two genomes, information about their alignment with a third genome does not affect synteny between the original pair of genomes. Thus our approach can be scaled to the problem of multiple genomes comparison without loss of precision. As a use case, given three genomes G_1,G_2,G_3, where G_1 is a reference genome, G_2 is a genome of interest, G_3 is an outgroup genome, we can build synteny blocks between pairs of $< G_1, G_2 >$ and $< G_1, G_3 >$ and assign evolutionary breakages of lineages of genomes G_1 and G_2 using G_3 as an outgroup.

halSynteny implements an algorithm for producing synteny blocks from genome alignment designed to process binary HAL files as input. The DAG-based method DAGchainer [4] was previously implemented for constructing synteny from the blast [23] alignments of gene annotations. It operates with homologous gene pairs found within complete genome sequences combining them into chains of syntenic genes. An alignment-based method SatsumaSynteny2 takes pairs of genome sequences as input and implements a dynamic programming algorithm for chaining the pairwise alignment blocks. Here we first apply the DAG-based approach to the whole-genome alignments. We define synteny for a pair of genomes aiming for more accurate results obtained from multiple genome alignment. Comparing to the other modern alignment-based software halSynteny allows for obtaining high-coverage results which follow from the definition of synteny. When comparing the performance of halSynteny to alignment-based software halSynteny produces much higher genome coverage, which agrees with the properties of the dataset. These results are closer to the results of anchor-based tools reported in the benchmark study [14], while halSynteny does not require intermediate genome annotation step. halSynteny can be installed as part of the halTools software essential for HAL-file processing and can be a useful tool when analyzing the whole-genome alignment data.

Availability of supporting source code and requirements

Project name: halSynteny
Project home page:
https://github.com/ComparativeGenomicsToolkit/hal
Operating system(s): Linux
Programming language: C++11
Other requirements: HAL API
License: MIT
RRID: SCR018127
biotoolsID: biotools:halSynteny

Funding

This publication was supported by a Subagreement from European Molecular Biology Laboratory with funds provided by Agreement No. 2U41HG007234-05 from National Institute of Health, NIGHR. It was also supported by the National Human Genome Research Institute of the National Institutes of Health under Award Number R01HG008742. Its content is solely the responsibility of the authors and do not necessarily represent the official views of National Institute of Health, NHGRI or European Molecular Biology Laboratory. Ksenia Krasheninnikova was supported by RFBR grant, project number 20-34-70055. Ksenia Krasheninnikova and Stephen O'Brien were supported, in part, by the Russian Science Foundation grant (project No. 17-14-01138) and by St. Petersburg State University (Genome Russia Grant no. 1.52.1647.2016).

Competing Interests

The author(s) declare that they have no competing interests.

Author's Contributions

Method development: K.K., M.D., J.A.; Implementation and testing: K.K., M.D., J.A.; Data preparation: K.K.; Supervision: M.D, B.P., S.J.B.; Definition of research project: K.K; Writing –

Table 1. Comparison of run time and genome coverage of resulting synteny blocks between SatsumaSynteny2 and halSynteny

Genome	Assembly N50 (Mbp)	Genome coverage (%)		Time required, min	
		SatsumaSynteny2	halSynteny	SatsumaSynteny2	halSynteny
S.ratti	11.7	55.6	72.5	1232	18(+496)
S. stercoralis	0.4	56.6	55.5		
C.elegans	17.5	20.0	92.5	547	74(+496)
C.briggsae	108.4	18.7	88.3		

Comparison of run time and genome coverage of resulting synteny blocks between SatsumaSynteny2 and halSynteny. The former was run with extra parameter -threads 10. The latter was run with resolution parameters –maxAnchorDistance 1000000 (1Mb), –minBlockSize 100000 (100Kb) for S.ratti/S.stercoralis, –maxAnchorDistance 1000000 (1Mb), –minBlockSize 1000000 (1Mb) for C.elegans/C.briggsae. As a preliminary step for application of halSynteny, the whole-genome alignment among all four genomes was constructed using Progressive Cactus software, which took 496 minutes. The assemblies of C.elegans and C.briggsae are of chromosomal level, while there are scaffold-level assemblies for S.ratti and S.stercoralis.

review and editing: K.K., M.D., A.D, B.P, S.J.B

Acknowledgements

We would like to thank reviewers Christophe Dessimoz and Michael Hiller for the valuable comments and suggestions that helped to improve the manuscript.

References

1. Hickey G, Paten B, Zerbino D, Haussler D. HAL: A hierarchical format for storing and analyzing multiple genome alignments. Bioinformatics 2013;.
2. Pevzner P, Tesler G. Genome rearrangements in mammalian evolution: lessons from human and mouse genomes. Genome Research 2003;.
3. Kent WJ, Baertsch R, Hinrichs A, Miller W, Haussler D. Evolution's cauldron: Duplication, deletion, and rearrangement in the mouse and human genomes. Proceedings of the National Academy of Sciences of the United States of America 2003;.
4. Haas BJ, Delcher AL, R WJ, Salzberg SL. DAGchainer: A tool for mining segmental genome duplications and synteny. Bioinformatics 2004;.
5. Grabherr MG, Russell P, Meyer M, Mauceli E, Alföldi J, di Palma F, et al. Genome-wide synteny through highly sensitive sequence alignment: Satsuma. Bioinformatics 2010;.
6. Wang Y, Tang H, Debarry JD, Tan X, Li J, Wang X, et al. MCScanX: A toolkit for detection and evolutionary analysis of gene synteny and collinearity. Nucleic Acid Research 2012;.
7. Proost S, Fostier J, De Witte D, Dhoedt B, Demeester P, Van De Peer Y, et al. i-ADHoRe 3.0-fast and sensitive detection of genomic homology in extremely large data sets. Nucleic Acid Research 2012;.
8. Drillon G, Carbone A, Fischer G. SynChro: A fast and easy tool to reconstruct and visualize synteny blocks along eukaryotic chromosomes. PLoS One 2014;.
9. Pham SK, Pevzner PA. DRIMM-Synteny: Decomposing genomes into evolutionary conserved segments. Bioinformatics 2014;.
10. Soderlund C, Bomhoff M, Nelson WM. SyMAP v3.4: A turnkey synteny system with application to plant genomes. Nucleic Acid Research 2011;.
11. Rödelsperger C, Dieterich C. CYNTENATOR: Progressive gene order alignment of 17 vertebrate genomes. PLoS One 2010;.
12. Lilue J, Doran AG, Fiddes IT, Abrudan M, Armstrong J, Bennett R, et al. Sixteen diverse laboratory mouse reference genomes define strain-specific haplotypes and novel functional loci. Nature Genetics 2018;.
13. Zhang G, Li C, Li Q, Li B, Larkin DM, Lee C, et al. Comparative genomics reveals insights into avian genome evolution and adaptation. Science 2014;.
14. Liu D, Hunt M, Tsai IJ. Inferring synteny between genome assemblies: A systematic evaluation. BMC Bioinformatics 2018;.
15. Dobrynin P, Liu S, Tamazian G, Xiong Z, Yurchenko AA, Krasheninnikova K, et al. Genomic legacy of the African cheetah, Acinonyx jubatus. Genome Biology 2015;.
16. Choo SW, Rayko M, Tan TK, Hari R, Komissarov A, Wee WY, et al. Pangolin genomes and the evolution of mammalian scales and immunity. Genome Research 2016;.
17. Paten B, Earl D, Nguye nN, Diekhans M, Zerbino D, Haussler D. Cactus: Algorithms for genome multiple sequence alignment. Genome Research 2011;.
18. Paten B, Diekhans M, Earl D, John JS, Ma J, Suh B, et al. Cactus graphs for genome comparisons. Computer Science 2010;.
19. Schlenoff C, Gruninger M. The Process Specification Language (PSL) Overview and Version 1.0 Specification. Technical Reports 2004;.
20. Yang F, Graphodatsky A, O'Brien P, Colabella A, Solanky N, Squire M, et al. Reciprocal chromosome painting illuminates the history of genome evolution of the domestic cat, dog and human. Chromosome Research 2000;.
21. Kent WJ, Sugnet CW, Furey TS, Roskin KM, Pringle TH, Zahler AM, et al. The human genome browser at UCSC. Genome Research 2002;.
22. Raney BJ, Dreszer TR, Barber GP, Clawson H, Fujita PA, Wang T, et al. Track data hubs enable visualization of user-defined genome-wide annotations on the UCSC Genome Browser. Bioinformatics 2014;.
23. Altschul SF, Gish W, Miller W, Myers EW, Lipman DJ. Basic local alignment search tool. Journal of Molecular Biology 1990;.

Supplementary Figure 1

Click here to
access/download;Manuscript;supplementary_figure_1.pdf

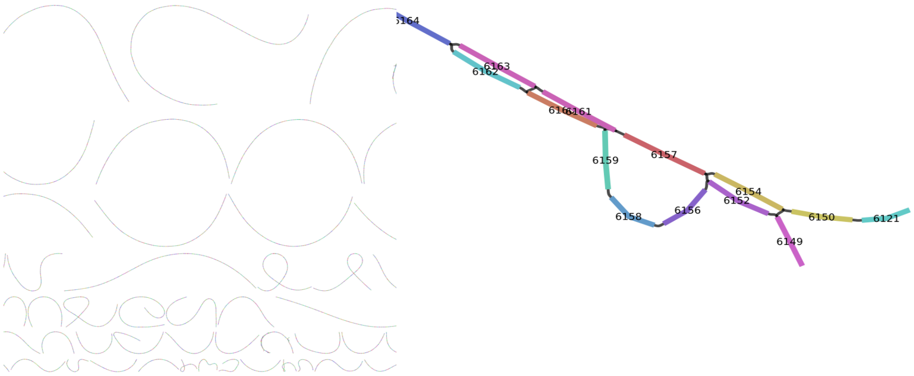

(a) Fragments of the graph reconstructed from the alignment blocks of the chromosome chr1 of S.ratti

(b) Detalization of the Figure 1a showing a local duplication (e.g. blocks 6152 and 6154)

Figure 1: DAG fragments corresponding to the comparison of S.ratti and S.stercoralis

Figure 1

Click here to access/download;Figure;figure1_cat_dog_red-

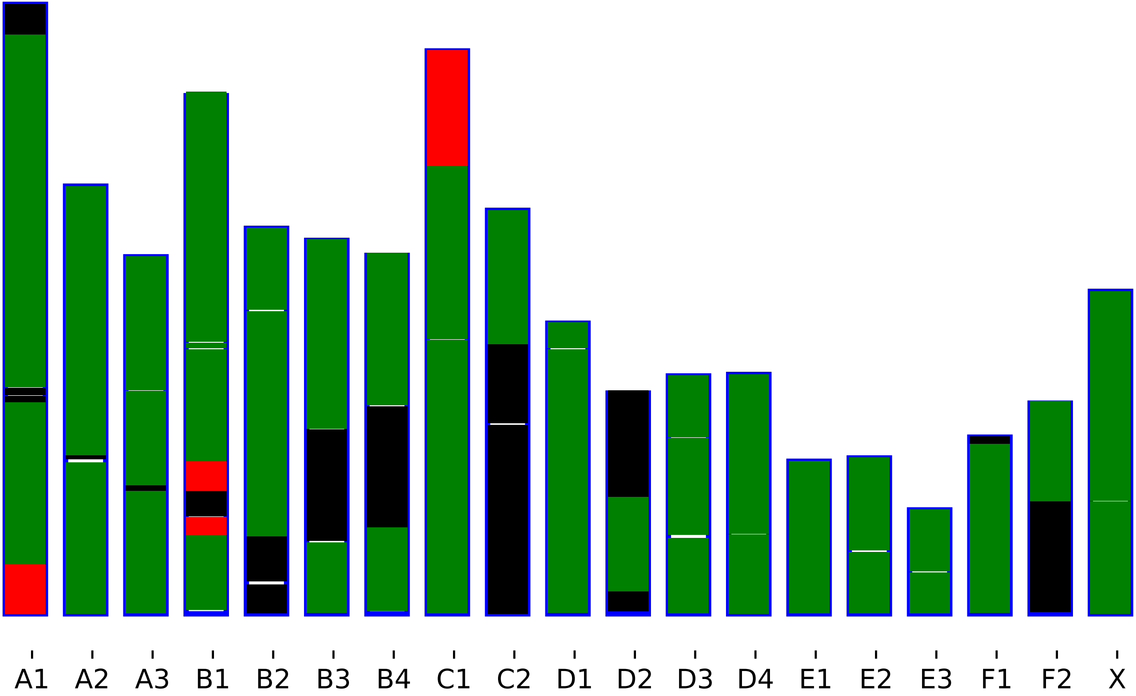

Supplement: giaa047_GIGA-D-19-00419_Revision_1 [file giaa047_giga-d-19-00419_revision_1.pdf]
