# Supplementary material for: halSynteny: a fast, easy-to-use conserved synteny block construction method for multiple whole-genome alignments
Source: Gigascience. 2020 May 28;9(6):giaa047. doi: 10.1093/gigascience/giaa047 (PMC7254927; doi:10.1093/gigascience/giaa047)
Supplement: giaa047_Supplemental_File [file giaa047_supplemental_file.pdf]

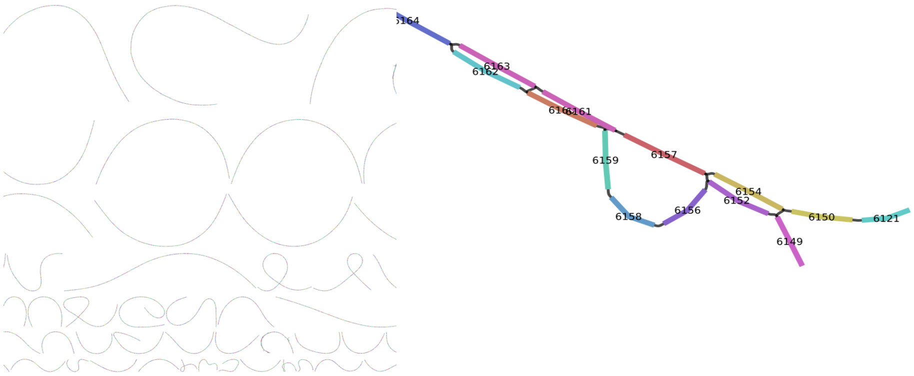

(a) Fragments of the graph reconstructed from the alignment blocks of the chromosome chr1 of S.ratti

(b) Detalization of the Figure 1a showing a local duplication (e.g. blocks 6152 and 6154)

Figure 1: DAG fragments corresponding to the comparison of S.ratti and S.stercoralis
