# Supplementary material for: halSynteny: a fast, easy-to-use conserved synteny block construction method for multiple whole-genome alignments
Source: Gigascience. 2020 May 28;9(6):giaa047. doi: 10.1093/gigascience/giaa047 (PMC7254927; doi:10.1093/gigascience/giaa047)
Supplement: giaa047_Response_to_Reviewer_Comments_Original_Submission [file giaa047_response_to_reviewer_comments_original_submission.pdf]

Author's Response To Reviewer Comments

Close

Dear Editor and Reviewers,
We do appreciate the reviewers' comments and their helpful suggestions for improving our manuscript. Based on these suggestions we included some elaborations into the text. We believe these changes have improved the quality and value of our work. A point-by-point response to the comments can be found below:
Reviewer #1:
This technical note describes a tool to compute synteny blocks from multiple genome alignments in HAL format, such as those produced by the tool Progressive Cactus.
The paper is a good fit to the editorial requirements of a technical note. The problem tackled is relevant. The algorithm is well described and makes sense. The code is available under an open access license.
I only have a couple of minor points that feel ought to be addressed before this paper can be accepted.
Minor points
- The documentation is very sparse. At the minimum, describe all the possible arguments.

Thank you. The documentation at github has been updated.

- Introduction: "There are a number of existing tools designed for finding synteny blocks [2, 3, 4, 5, 6, 7, 8, 9, 10]". It would be more reader-friendly if the authors provided a bit more context on these various citations.

We extended the description of the available software in the area of synteny blocks construction in the text.

- "Due to the modeling of duplications, different alignment blocks can overlap in coordinates in either genome.". Provide more explanations about how duplications are handled, as this is central to this problem.

Each gapless alignment block between the pair of genomes is represented with the start and end positions on the chromosomes, along with the strand. Duplications are expressed as overlapping alignment blocks. Presume there are alignment hits in genome A that can be ordered by genomic coordinates as p1 ···pi,···pn and in the genome B as u1 ···ui,ui+1,···un+1, and there are alignments present among segments pl and ul for l ∈ 1 · · · i pk and uk+1 for k ∈ i · · · n. Then the synteny blocks between the genomes A and B can overlap and contain the following pairs of segments: (pi, ui) for l ∈ 1 · · · i and (pk, uk+1) for k ∈ i···n.
We included this explanation into the text of the manuscript. Also, please, see Supplementary Figure 1 for illustration of handling duplications.

- Differences between halSynteny and chromosomal painting in red segments A1, B1, and C1. It should be possible to resolve the discrepancy. I had a look at the synteny dot plot in the OMA database, where orthology is inferred at the level of individual genes, and it appears that all three are syntenic with dog chromosome 25 and not dog chromosome 28.
orthologous segments here:
https://omabrowser.org/oma/dotplot/FELCA/CANLF/A1/25/
https://omabrowser.org/oma/dotplot/FELCA/CANLF/B1/25/
https://omabrowser.org/oma/dotplot/FELCA/CANLF/C1/25/
No orthology here:
https://omabrowser.org/oma/dotplot/FELCA/CANLF/A1/28/
https://omabrowser.org/oma/dotplot/FELCA/CANLF/B1/28/
https://omabrowser.org/oma/dotplot/FELCA/CANLF/C1/28/

We thank the Reviewer for this valuable addition to the question of contradictory analysis of synteny for the dog chromosomes 25 and 28. This valuable evidence supports the conclusions based on the whole-genome alignments described in the manuscript.

Discretionary:
- The algorithm is provided without proof on correctness, or analysis on asymptotic time and space costs. Perhaps worth including these if you have them or can do them easily.

We thank the Reviewer for this valuable remark, as it’s worth emphasizing that the algorithm is heuristical. In the course of the problem solution we desire to achieve two goals: the desired set of synteny blocks should contain as many con- tinuous synteny blocks as possible covering as much of both genome sequences as possible. Although we do not provide a strict mathematical proof of conditions which would always allow that both goals are achievable simultaneously, we describe the heuristic that strives to achieve them. The manuscript text is updated with more detailed definitions. In the implementation of this algorithm available on github we store the graph as adjacency list which allows for asymptotic time and space complexity $O(|V| + |E|)$.

Typos
- Ref. 19 seems not to be the correct one for chromosomal painting. Thank you. It’s fixed.
- Italicise species names in Table 1.

Fixed.

- On the GitHub README file, ”Genom1” → ”Genome1”

Thank you. Fixed.

Reviewer #2:
This manuscript describes a new tool, HalSynteny, that uses a dynamic programming algorithm to extract maximal pairwise syntenic regions from pairwise or multiple whole genome alignments. The tool is substantially faster than a previous method and extracts much larger syntenic regions. Thus, HalSynteny will likely have broad applicability.
I have only a few comments that I hope are helpful, and some questions.
1) The initial weight of vertex wvi is the length of the ungapped alignment block. I was wondering why not the alignment score (e.g. using a simple scoring scheme like +1 for a match as in Blat) is chosen. If there are two ungapped alignment blocks of equal size but one with a higher identity (thus alignment score), it would be preferable to include this block in the syntenic block.

We thank Professor Hiller for the reasonable suggestion. In fact, the goal was to find the largest continuous regions, while the identification of differences was not in scope. Here we rely on the alignment which has already established the most probable ordering.

2) In my printout, I can’t really see the blue lines in figure 1. Also, the text writes about ’purple regions’ but I think this is indicated as black in the figure. The blue lines refer to the contour of the schematic representation of chromosome on the Figure 1 in main text. We updated the text with regard to this concern.

We apologize for the misprint, since the ’purple regions’ should be ’red regions’. This is corrected in the manuscript.

3) While I believe that HalSynteny is much more accurate than chromosome painting, also because it is based on actual sequence alignments, I was wonder- ing what could possibly explain the large difference between both methods for the cat chromosomes A1, B1, C1.

There are studies [1], [2] describing possibilities and limitations of the chromosomal painting approach. Although this approach provides an efficient technique to discover large-scale similarity of continuous homology, it tends to misclassify small insertions and small translocations [1]. Also, it was reported that in cases of complex rearrangements chromosomal painting can be laborious and requires confirmation [2]. Our results supported by the orthologous alignments kindly provided by the Reviewer 1 advocate that the identified blocks of chromosomes A1, B1, and C1 are syntenic to the dog chromosome 25. Lastly, the synteny parameters (bmin, dmax) used were both bmin and dmax were set as 1Mb. This has been inserted into the text.

4) The authors could discuss in the text that HalSynteny is substantially faster than SatsumaSynteny2. I assume here that the runtime of SatsumaSynteny2 also does not include the time needed to produce

the input genome alignment. Otherwise, this should be included in HalSynteny or excluded in SatsumaSynteny2 to make the runtime comparable.

Indeed, the alignment procedure requires most significant portion of time in both approaches to the synteny block construction. However in case of halSynteny, which uses alignment as input, the alignment procedure is self-sufficient and the produced output can be used independently for other analysis. SatsumaSynteny2 operates over fasta files as input and produces internal alignment which is not separable from results that's why the timing for SatsumaSynteny2 includes also alignment step. In this manuscript to benchmark halSynteny by construct- ing blocks between S.ratti and S.stercoralis, and separately for C.elegans and C.briggsae, we performed one alignment of all 4 species which took 496 minutes. After that running halSynteny over the alignment took 18 min and 74 min. We understand some uncertainty which arises from different approaches and added the alignment time in parentheses to the table along with its mentioning in the table's caption.

5) There are substantial differences in the coverage of syntenic blocks between both methods. Is this due to different synteny parameters? E.g. is SatsumaSynteny2 requiring much larger and more stringent syntenic blocks? If I understand the HalSynteny algorithm correctly, a syntenic block can 'skip over' a local inversion or local duplication without breaking the syntenic block. Maybe SatsumaSynteny2 does not possess this feature and stops at local rearrangements, which could explain these performance differences.

We used the comparison protocol described in [3]. The authors also observe that "Satsuma demonstrates fewer contiguation-dependent patterns as its detection of synteny relies on nucleotide alignments". This hypothesis may not be correct since our algorithm also employs nucleotide alignment but demonstrates higher coverage on the same data. Satsuma implements a completely different method based on the Fast Furier transform over the signal represented by nucleotide multiplication pattern. We can assume that the internal parameters limiting the enough bounds for the signal affect the coverage of synteny blocks.

Other suggestions: - I would merge the 'Finally it allows ..." sentence with the previous paragraph.

Thank you. Done.

- Discussion: While not the focus of the paper, I wonder whether a < G2,G3 > comparison would be necessary to detect breakages that occurred in the lineage leading to G2 (I think this is not detectable from a < G1, G2 > and < G1, G3 > comparison).

Usually an outgroup genome is selected for assigning breakpoints to G1 or G2, if we would like to consider breakages between G2 and G3 we would need to make a comparison < G2,G3 > as well as to use another genome G4 evolutionary distant enough from both as an outgroup.

- As a suggestion, a figure sketch illustrating the graph and a maximal syntenic block would be helpful. Maybe one could illustrate a case of skipping over a local inversion or duplication.

Thank you for this suggestion. Supplementary Figure 1 illustrates the process of synteny block construction. The alignment blocks for each query chromosome are numbered according to their appearance in the alignment traversal. Blocks 6152 and 6154 in the Supplemetary Figure 1b correspond to a genomic duplication. The algorithm described in the manuscript constructed a synteny block A from alignment blocks 6161,6157, 6152, 6149, etc. Depending on the threshold parameter regulating the minimal size of a synteny block alignment blocks 6121, 6150, 6154 could consititute another synteny block B which would overlap in genomic coordinates with A. If the genomic size of the region corresponding to blocks 6121, 6150, 6154 is less than the defined parameter, then the corresponding genomic regions won't be covered by synteny blocks.
Supplementary Figure 1 is obtained using Bandage software [4].

- Assembly N50 probably refers to scaffold N50?

Thank you for this remark. The assemblies of C.elegans and C.briggsae are of chromosomal level, while there are scaffold-level assemblies for S.ratti and S.stercoralis. This has been added to the main text.

- Michael Hiller

References
[1] C. Lee et al. “Limitations of Chromosome Classification by Multicolor Karyotyping”. In: AJHG (2001).
[2] T. Ried et al. “Chromosome Painting: A Useful Art”. In: Human Molecular Genetics (1998).
[3] D. Liu and I. J. Hunt M.and Tsai. “Inferring synteny between genome assemblies: A systematic evaluation”. In: BMC Bioinformatics (2018).
[4] R.R. Wick et al. “Bandage: interactive visualisation of de novo genome assemblies”. In: Bioinformatics (2015).

Close
