# Supplementary material for: halSynteny: a fast, easy-to-use conserved synteny block construction method for multiple whole-genome alignments
Source: Gigascience. 2020 May 28;9(6):giaa047. doi: 10.1093/gigascience/giaa047 (PMC7254927; doi:10.1093/gigascience/giaa047)
Supplement: giaa047_Response_to_Reviewer_Comments_Revision_1 [file giaa047_response_to_reviewer_comments_revision_1.pdf]

Author's Response To Reviewer Comments

Close

Dear Editor and Reviewer,

We are very grateful for your comments and suggestions. The revised version of the manuscript contains additional text describing where the differences in chromosomal painting and halSynteny approaches can stem from. We also added description of the gene-level orthology evidence and the corresponding references to the orthology plots kindly provided by the Reviewer #1 into the referenced GigaDB dataset.

Best regards,

Ksenia Krasheninnikova

Close
