# Supplementary material for: halSynteny: a fast, easy-to-use conserved synteny block construction method for multiple whole-genome alignments
Source: Gigascience. 2020 May 28;9(6):giaa047. doi: 10.1093/gigascience/giaa047 (PMC7254927; doi:10.1093/gigascience/giaa047)
Supplement: giaa047_Reviewer_2_Report_Original_Submission — Michael Hiller -- 1/11/2020 Reviewed [file giaa047_reviewer_2_report_original_submission.pdf]

Reviewer Report

Title: halSynteny: a fast, easy-to-use conserved synteny block construction method for multiple whole-genome alignments

Version: Original Submission **Date:** 1/11/2020

Reviewer name: Michael Hiller

Reviewer Comments to Author:

This manuscript describes a new tool, HalSynteny, that uses a dynamic programming algorithm to extract maximal pairwise syntenic regions from pairwise or multiple whole genome alignments. The tool is substantially faster than a previous method and extracts much larger syntenic regions. Thus, HalSynteny will likely have broad applicability.
I have only a few comments that I hope are helpful, and some questions.
1) The initial weight of vertex wvi is the length of the ungapped alignment block. I was wondering why not the alignment score (e.g. using a simple scoring scheme like +1 for a match as in Blat) is chosen. If there are two ungapped alignment blocks of equal size but one with a higher identity (thus alignment score), it would be preferable to include this block in the syntenic block.
2) In my printout, I can't really see the blue lines in figure 1. Also, the text writes about 'purple regions' but I think this is indicated as black in the figure.
3) While I believe that HalSynteny is much more accurate than chromosome painting, also because it is based on actual sequence alignments,
I was wondering what could possibly explain the large difference between both methods for the cat chromosomes A1, B1, C1.
Also, the synteny parameters (bmin, dmax) used for the cat/dog comparison should be mentioned in the text.
4) The authors could discuss in the text that HalSynteny is substantially faster than SatsumaSynteny2. I assume here that the runtime of SatsumaSynteny2 also does not include the time needed to produce the input genome alignment. Otherwise, this should be included in HalSynteny or excluded in SatsumaSynteny2 to make the runtime comparable.
5) There are substantial differences in the coverage of syntenic blocks between both methods. Is this due to different synteny parameters? E.g. is SatsumaSynteny2 requiring much larger and more stringent syntenic blocks?
If I understand the HalSynteny algorithm correctly, a syntenic block can 'skip over' a local inversion or local duplication without breaking the syntenic block. Maybe SatsumaSynteny2 does not possess this feature and stops at local rearrangements, which could explain these performance differences.
Other suggestions:
- I would merge the 'Finally it allows ..." sentence with the previous paragraph.
- Discussion: While not the focus of the paper, I wonder whether a <g2,g3> comparison would be necessary to detect breakages that occurred in the lineage leading to G2 (I think this is not detectable from a <g1,g2> and <g1,g3> comparison).

- As a suggestion, a figure sketch illustrating the graph and a maximal syntenic block would be helpful. Maybe one could illustrate a case of skipping over a local inversion or duplication.
- Assembly N50 probably refers to scaffold N50?
- Michael Hiller

Level of Interest

Please indicate how interesting you found the manuscript: Choose an item.

Quality of Written English

Please indicate the quality of language in the manuscript: Choose an item.

Declaration of Competing Interests

Please complete a declaration of competing interests, considering the following questions:

- Have you in the past five years received reimbursements, fees, funding, or salary from an organisation that may in any way gain or lose financially from the publication of this manuscript, either now or in the future?
- Do you hold any stocks or shares in an organisation that may in any way gain or lose financially from the publication of this manuscript, either now or in the future?
- Do you hold or are you currently applying for any patents relating to the content of the manuscript?
- Have you received reimbursements, fees, funding, or salary from an organization that holds or has applied for patents relating to the content of the manuscript?
- Do you have any other financial competing interests?
- Do you have any non-financial competing interests in relation to this paper?

If you can answer no to all of the above, write 'I declare that I have no competing interests' below. If your reply is yes to any, please give details below.

None

I agree to the open peer review policy of the journal. I understand that my name will be included on my report to the authors and, if the manuscript is accepted for publication, my named report including any attachments I upload will be posted on the website along with the authors' responses. I agree for my report to be made available under an Open Access Creative Commons CC-BY license (http://creativecommons.org/licenses/by/4.0/). I understand that any comments which I do not wish to be included in my named report can be included as confidential comments to the editors, which will not be published.

Choose an item.

To further support our reviewers, we have joined with Publons, where you can gain additional credit to further highlight your hard work (see: https://publons.com/journal/530/gigascience). On publication of this paper, your review will be automatically added to Publons, you can then choose whether or not to claim your Publons credit. I understand this statement.

Yes Choose an item.
